# 1-(2,6-Dichloro­benzo­yl)-3-(3-methoxy­phen­yl)thio­urea

**DOI:** 10.1107/S1600536808041263

**Published:** 2008-12-17

**Authors:** M. Khawar Rauf, Michael Bolte, Amin Badshah

**Affiliations:** a Department of Chemistry, Quaid-i-Azam University Islamabad, 45320-Pakistan; b Institut für Anorganische Chemie, J. W. Goethe-Universität Frankfurt, Max-von-Laue-Str. 7, 60438 Frankfurt/Main, Germany

## Abstract

The two aromatic rings in the title compound, C_15_H_12_Cl_2_N_2_O_2_S, enclose a dihedral angle of 37.49 (6)°. The mol­ecule exists in the solid state in its thione form with typical thio­urea C—S and C—O bonds lengths, as well as shortened C—N bonds. An intra­molecular N—H⋯O hydrogen bond stabilizes the mol­ecular conformation. In the crystal, mol­ecules are connected by N—H⋯O and N—H⋯S hydrogen bonds, forming chains running along the *a* axis.

## Related literature

For general background, see: Darlington *et al.* (1996 ▶); Dowding & Leeds (1971 ▶); Sasse *et al.* (1969 ▶); Khawar Rauf *et al.*, (2006*a* ▶,*b* ▶,*c* ▶, 2007 ▶); Santrucek & Krepelka (1988 ▶); Teruhisa *et al.* (1972 ▶). For related structures, see: Khawar Rauf *et al.* (2006*a* ▶,*b* ▶,*c* ▶, 2007 ▶). For a description of the Cambridge Database, see: Allen, (2002 ▶).
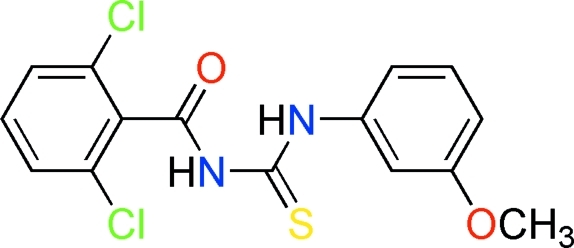


## Experimental

### 

#### Crystal data

C_15_H_12_Cl_2_N_2_O_2_S
*M* *_r_* = 355.23
Monoclinic, 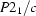

*a* = 10.7215 (6) Å
*b* = 11.2370 (8) Å
*c* = 13.8065 (8) Å
β = 104.447 (4)°
*V* = 1610.77 (17) Å^3^
*Z* = 4
Mo *K*α radiation
μ = 0.54 mm^−1^
*T* = 173 (2) K
0.47 × 0.47 × 0.44 mm

#### Data collection

STOE IPDS II two-circle-diffractometer
Absorption correction: multi-scan (*MULABS*; Spek, 2003 ▶; Blessing, 1995 ▶) *T*_min_ = 0.786, *T*_max_ = 0.797
19919 measured reflections
4113 independent reflections
3704 reflections with *I* > 2σ(*I*)
*R*_int_ = 0.060

#### Refinement

*R*[*F* ^2^ > 2σ(*F* ^2^)] = 0.035
*wR*(*F* ^2^) = 0.087
*S* = 1.07
4113 reflections
218 parameters
2 restraints
H atoms treated by a mixture of independent and constrained refinement
Δρ_max_ = 0.33 e Å^−3^
Δρ_min_ = −0.33 e Å^−3^

### 

Data collection: *X-AREA* (Stoe & Cie, 2001 ▶); cell refinement: *X-AREA*; data reduction: *X-AREA*; program(s) used to solve structure: *SHELXS97* (Sheldrick, 2008 ▶); program(s) used to refine structure: *SHELXL97* (Sheldrick, 2008 ▶); molecular graphics: *XP* in *SHELXTL-Plus* (Sheldrick, 2008 ▶); software used to prepare material for publication: *SHELXL97*.

## Supplementary Material

Crystal structure: contains datablocks I, global. DOI: 10.1107/S1600536808041263/dn2414sup1.cif

Structure factors: contains datablocks I. DOI: 10.1107/S1600536808041263/dn2414Isup2.hkl

Additional supplementary materials:  crystallographic information; 3D view; checkCIF report

## Figures and Tables

**Table 1:** Hydrogen-bond geometry (Å, °)

*D*—H⋯*A*	*D*—H	H⋯*A*	*D*⋯*A*	*D*—H⋯*A*
N2—H2⋯O1	0.89 (2)	1.97 (2)	2.7007 (15)	137.9 (18)
N2—H2⋯O1^i^	0.89 (2)	2.38 (2)	3.1015 (16)	137.8 (18)
N1—H1⋯S1^ii^	0.852 (18)	2.479 (19)	3.3141 (12)	166.7 (16)

Symmetry codes: (i) 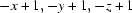; (ii) 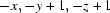.

**Fig. 1.**
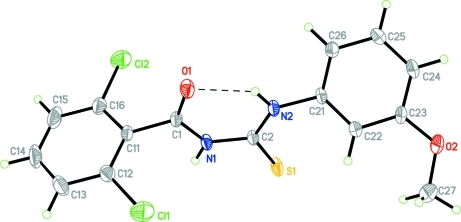
Molecular structure of (I) with the atom-labeling scheme. Displacement ellipsoids are drawn at the 50% probability level. Hydrogen bond is shown as dashed lines.

**Fig. 2.**
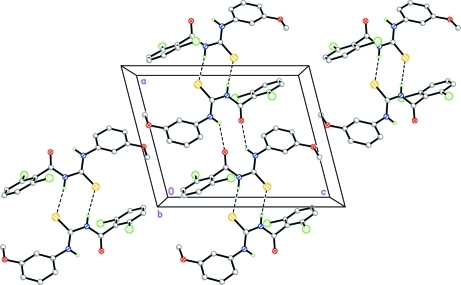
Partial packing view of (I) onto the ac plane. H atoms not involved in hydrogen bonding are omitted. Hydrogen bonds are shown as dashed lines.

## supplementary crystallographic information

### Comment

Thiourea and Urea derivatives have played an important role in developing agrochemicals and pharmacological agents, for example. Ureidothiazoles are effective herbicides for a broad spectrum of weeds (Dowding & Leeds, 1971). *N*-Methylfurfurylurea herbicides gave selective weed control in cereals, as well as cotton and beans (Sasse *et al.*, 1969). *N*-Methyl-*N*-(2-thiazolyl)-*N'*-alkyl substituted thioureas are plant growth regulators that inhibit stem alongation of rice and kidney bean plants without phytotoxity (Teruhisa *et al.*, 1972). 4-Aminomethyl derivatives of 2-methyl-5-hydroxybenzimidazole have been reported as an antioxidants and stimmulators of plant growth of dicotyledons under drought conditions (Santrucek & Krepelka, 1988; Darlington *et al.*, 1996). As part of interest in *N*,*N'*-disubstituted thioureas, we now report the crystal structure of the title compound (I).

A view of compound (I), is shown in Fig 1. The N—C bonds differ significantly from one another but are short in comparison with the typical value for an N—C single bond (1.479 A°), and the C1—S1 bond is slightly shorter than a C—S double bond (1.681 A°), indicating partial electron delocalization in the N—C(S)—N(H)—C(O) structural segment. These distances are similar to those usually found in other substituted thioureas [Khawar Rauf *et al.*, 2006*a*, 2006*b*, 2006*c*, 2007; Cambridge Structural Database, Version 5.28 (Allen, 2002)]. The dihedral angle between the aromatic rings is 37.49 (6)°, and the corresponding angles with the thiourea plane are 81.41 (7)° for the C11–C16 ring and 44.12 (7)° for the C21–C26 ring. The thiocarbonyl and carbonyl groups are almost coplanar, as reflected by the O1—C2—N2—C1 and C2—N2— C1—N1 torsion angles. This is associated with the intramolecular N—H···O hydrogen bond (Table 1), forming a six-membered ring commonly observed in this class of compounds. In the crystal packing of (I), intermolecular N—H···S and N-H···O hydrogen bonds link the molecules into chains running along the *a* axis (Table 1, Fig. 2).

### Experimental

Freshly prepared 2,6-Dichlorobenzoylisothiocyanate (2.32 g, 10 mmol) was added in acetone (30 ml) and stirred for 10 minutes. Afterwards neat 3-methoxyaniline (1.23 g, 10 mmol) was added and the resulting mixture was stirred for 1 h.The reaction mixture was then poured into acidified (pH 4) water and stirred well. The solid product was separated and washed with deionized water and purified by recrystallization from methanol/ 1,1-dichloromethane (1:1 *v/v*) to give fine crystals of the title compound (I), with an overall yield of 88%. Full spectroscopic and physical characterization will be reported elsewhere.

### Refinement

Hydrogen atoms bonded to C were included in calculated positions and refined as riding on their parent C atom with C—H = 0.95 Å *U*_iso_(H) = 1.2U(C_eq_) or C—H = 0.98 Å and *U*_iso_(H) = 1.5U(C_eq_), respectively, for aromatic and methyl C atoms. The methyl group is disordered over two positions with site occupation factors 0.76 (3) and 0.24 (3). For refinement the *O*—C_methyl_ and C_aromatic_^···^C_methyl_ distances were restrained to be equal with an effective standard deviation of 0.02 Å. H atoms bonded to N were refined freely.

### Crystal data

**Table:**

C_15_H_12_Cl_2_N_2_O_2_S	*F*(000) = 728
*M**_r_* = 355.23	*D*_x_ = 1.465 Mg m^−^^3^
Monoclinic, *P*2_1_/*c*	Mo *K*α radiation, λ = 0.71073 Å
Hall symbol: -P 2ybc	Cell parameters from 7361 reflections
*a* = 10.7215 (6) Å	θ = 3.5–28.2°
*b* = 11.2370 (8) Å	µ = 0.54 mm^−^^1^
*c* = 13.8065 (8) Å	*T* = 173 K
β = 104.447 (4)°	Block, colourless
*V* = 1610.77 (17) Å^3^	0.47 × 0.47 × 0.44 mm
*Z* = 4	

### Data collection

**Table:**

STOE IPDS II two-circle-diffractometer	4113 independent reflections
Radiation source: fine-focus sealed tube	3704 reflections with *I* > 2σ(*I*)
graphite	*R*_int_ = 0.060
ω scans	θ_max_ = 28.6°, θ_min_ = 3.6°
Absorption correction: multi-scan (*MULABS*; Spek, 2003; Blessing, 1995)	*h* = −13→14
*T*_min_ = 0.786, *T*_max_ = 0.797	*k* = −15→15
19919 measured reflections	*l* = −18→18

### Refinement

**Table:**

Refinement on *F*^2^	Secondary atom site location: difference Fourier map
Least-squares matrix: full	Hydrogen site location: inferred from neighbouring sites
*R*[*F*^2^ > 2σ(*F*^2^)] = 0.035	H atoms treated by a mixture of independent and constrained refinement
*wR*(*F*^2^) = 0.087	*w* = 1/[σ^2^(*F*_o_^2^) + (0.0324*P*)^2^ + 0.7918*P*] where *P* = (*F*_o_^2^ + 2*F*_c_^2^)/3
*S* = 1.07	(Δ/σ)_max_ < 0.001
4113 reflections	Δρ_max_ = 0.33 e Å^−^^3^
218 parameters	Δρ_min_ = −0.33 e Å^−^^3^
2 restraints	Extinction correction: *SHELXL*, Fc^*^=kFc[1+0.001xFc^2^λ^3^/sin(2θ)]^-1/4^
Primary atom site location: structure-invariant direct methods	Extinction coefficient: 0.0178 (13)

### Special details

**Table:**

Geometry. All e.s.d.'s (except the e.s.d. in the dihedral angle between two l.s. planes) are estimated using the full covariance matrix. The cell e.s.d.'s are taken into account individually in the estimation of e.s.d.'s in distances, angles and torsion angles; correlations between e.s.d.'s in cell parameters are only used when they are defined by crystal symmetry. An approximate (isotropic) treatment of cell e.s.d.'s is used for estimating e.s.d.'s involving l.s. planes.
Refinement. Refinement of *F*^2^ against ALL reflections. The weighted *R*-factor *wR* and goodness of fit *S* are based on *F*^2^, conventional *R*-factors *R* are based on *F*, with *F* set to zero for negative *F*^2^. The threshold expression of *F*^2^ > σ(*F*^2^) is used only for calculating *R*-factors(gt) *etc*. and is not relevant to the choice of reflections for refinement. *R*-factors based on *F*^2^ are statistically about twice as large as those based on *F*, and *R*- factors based on ALL data will be even larger.

### Fractional atomic coordinates and isotropic or equivalent isotropic displacement parameters (Å^2^)

**Table:**

	*x*	*y*	*z*	*U*_iso_*/*U*_eq_	Occ. (<1)
S1	0.10910 (3)	0.40403 (4)	0.62296 (3)	0.02598 (11)	
Cl1	0.19499 (5)	0.79513 (4)	0.43009 (3)	0.03990 (12)	
Cl2	0.19839 (4)	0.38864 (4)	0.22155 (3)	0.03747 (12)	
O1	0.37925 (9)	0.51869 (10)	0.42963 (7)	0.0259 (2)	
O2	0.41731 (16)	0.32269 (15)	0.95663 (9)	0.0556 (4)	
N1	0.18818 (11)	0.50043 (11)	0.47460 (8)	0.0194 (2)	
H1	0.1077 (18)	0.5144 (16)	0.4543 (13)	0.021 (4)*	
N2	0.35139 (11)	0.41869 (11)	0.60087 (8)	0.0190 (2)	
H2	0.400 (2)	0.4382 (19)	0.5596 (16)	0.038 (5)*	
C1	0.26337 (12)	0.53548 (12)	0.41218 (9)	0.0177 (2)	
C2	0.22582 (12)	0.44147 (12)	0.56675 (9)	0.0175 (2)	
C11	0.18968 (12)	0.59740 (12)	0.31805 (9)	0.0179 (2)	
C12	0.15568 (14)	0.71706 (13)	0.31802 (11)	0.0238 (3)	
C13	0.09316 (16)	0.77639 (16)	0.23118 (13)	0.0348 (4)	
H13	0.0715	0.8582	0.2331	0.042*	
C14	0.06301 (18)	0.71414 (19)	0.14161 (13)	0.0419 (5)	
H14	0.0199	0.7535	0.0817	0.050*	
C15	0.09516 (17)	0.59545 (18)	0.13874 (11)	0.0377 (4)	
H15	0.0740	0.5534	0.0771	0.045*	
C16	0.15873 (14)	0.53731 (14)	0.22639 (10)	0.0241 (3)	
C21	0.41141 (12)	0.35406 (12)	0.69003 (9)	0.0179 (2)	
C22	0.38111 (14)	0.37739 (14)	0.78086 (10)	0.0247 (3)	
H22	0.3202	0.4371	0.7856	0.030*	
C23	0.44246 (16)	0.31089 (15)	0.86431 (10)	0.0294 (3)	
C24	0.53374 (15)	0.22526 (14)	0.85823 (11)	0.0285 (3)	
H24	0.5747	0.1802	0.9156	0.034*	
C25	0.56442 (15)	0.20629 (14)	0.76804 (11)	0.0267 (3)	
H25	0.6280	0.1489	0.7640	0.032*	
C26	0.50342 (13)	0.27027 (13)	0.68283 (10)	0.0219 (3)	
H26	0.5246	0.2566	0.6209	0.026*	
C27	0.3347 (12)	0.4200 (12)	0.9700 (4)	0.067 (2)	0.76 (3)
H27A	0.3239	0.4185	1.0384	0.101*	0.76 (3)
H27B	0.2505	0.4117	0.9224	0.101*	0.76 (3)
H27C	0.3739	0.4957	0.9582	0.101*	0.76 (3)
C27'	0.293 (2)	0.374 (2)	0.9566 (14)	0.050 (4)	0.24 (3)
H27D	0.2833	0.3776	1.0254	0.075*	0.24 (3)
H27E	0.2241	0.3237	0.9163	0.075*	0.24 (3)
H27F	0.2864	0.4540	0.9282	0.075*	0.24 (3)

### Atomic displacement parameters (Å^2^)

**Table:**

	*U*^11^	*U*^22^	*U*^33^	*U*^12^	*U*^13^	*U*^23^
S1	0.01571 (15)	0.0392 (2)	0.02369 (18)	0.00331 (13)	0.00612 (12)	0.01717 (14)
Cl1	0.0503 (3)	0.0295 (2)	0.0402 (2)	−0.00054 (17)	0.01176 (19)	−0.00903 (16)
Cl2	0.0458 (2)	0.0322 (2)	0.0353 (2)	0.00040 (17)	0.01168 (17)	−0.00861 (15)
O1	0.0145 (4)	0.0418 (6)	0.0210 (5)	0.0029 (4)	0.0040 (4)	0.0109 (4)
O2	0.0727 (10)	0.0803 (11)	0.0164 (5)	0.0448 (9)	0.0159 (6)	0.0154 (6)
N1	0.0129 (5)	0.0287 (6)	0.0162 (5)	0.0031 (4)	0.0026 (4)	0.0089 (4)
N2	0.0150 (5)	0.0272 (6)	0.0143 (5)	0.0021 (4)	0.0025 (4)	0.0068 (4)
C1	0.0167 (6)	0.0219 (6)	0.0137 (5)	0.0000 (5)	0.0023 (4)	0.0031 (4)
C2	0.0165 (5)	0.0202 (6)	0.0147 (5)	0.0010 (5)	0.0021 (4)	0.0038 (4)
C11	0.0149 (5)	0.0241 (6)	0.0147 (6)	0.0004 (5)	0.0038 (4)	0.0054 (5)
C12	0.0217 (6)	0.0264 (7)	0.0249 (7)	0.0025 (5)	0.0086 (5)	0.0061 (5)
C13	0.0310 (8)	0.0355 (9)	0.0403 (9)	0.0124 (7)	0.0133 (7)	0.0202 (7)
C14	0.0357 (9)	0.0623 (12)	0.0267 (8)	0.0154 (8)	0.0057 (7)	0.0253 (8)
C15	0.0359 (8)	0.0600 (12)	0.0147 (6)	0.0045 (8)	0.0013 (6)	0.0056 (7)
C16	0.0228 (6)	0.0306 (7)	0.0182 (6)	0.0002 (6)	0.0040 (5)	0.0018 (5)
C21	0.0157 (5)	0.0216 (6)	0.0141 (5)	0.0001 (5)	−0.0004 (4)	0.0052 (4)
C22	0.0259 (7)	0.0298 (7)	0.0175 (6)	0.0089 (6)	0.0036 (5)	0.0047 (5)
C23	0.0335 (8)	0.0400 (9)	0.0135 (6)	0.0107 (7)	0.0039 (5)	0.0058 (6)
C24	0.0307 (7)	0.0323 (8)	0.0199 (6)	0.0084 (6)	0.0011 (6)	0.0099 (6)
C25	0.0260 (7)	0.0276 (7)	0.0255 (7)	0.0094 (6)	0.0045 (6)	0.0063 (5)
C26	0.0215 (6)	0.0263 (7)	0.0175 (6)	0.0027 (5)	0.0042 (5)	0.0033 (5)
C27	0.078 (4)	0.100 (5)	0.0266 (15)	0.053 (4)	0.018 (2)	0.004 (2)
C27'	0.075 (9)	0.060 (9)	0.024 (6)	0.031 (7)	0.030 (6)	0.015 (5)

### Geometric parameters (Å, °)

**Table:**

S1—C2	1.6822 (13)	C14—H14	0.9500
Cl1—C12	1.7367 (16)	C15—C16	1.394 (2)
Cl2—C16	1.7293 (16)	C15—H15	0.9500
O1—C1	1.2198 (16)	C21—C26	1.3852 (19)
O2—C23	1.3735 (17)	C21—C22	1.3965 (18)
O2—C27'	1.454 (12)	C22—C23	1.3930 (19)
O2—C27	1.448 (5)	C22—H22	0.9500
N1—C1	1.3763 (16)	C23—C24	1.390 (2)
N1—C2	1.4015 (16)	C24—C25	1.382 (2)
N1—H1	0.852 (18)	C24—H24	0.9500
N2—C2	1.3355 (16)	C25—C26	1.3946 (19)
N2—C21	1.4358 (15)	C25—H25	0.9500
N2—H2	0.89 (2)	C26—H26	0.9500
C1—C11	1.5116 (17)	C27—H27A	0.9800
C11—C12	1.393 (2)	C27—H27B	0.9800
C11—C16	1.3993 (19)	C27—H27C	0.9800
C12—C13	1.389 (2)	C27'—H27D	0.9800
C13—C14	1.387 (3)	C27'—H27E	0.9800
C13—H13	0.9500	C27'—H27F	0.9800
C14—C15	1.381 (3)		
			
C23—O2—C27'	115.5 (7)	C11—C16—Cl2	119.74 (11)
C23—O2—C27	117.3 (3)	C26—C21—C22	121.43 (12)
C1—N1—C2	128.51 (11)	C26—C21—N2	117.20 (11)
C1—N1—H1	116.4 (12)	C22—C21—N2	121.34 (12)
C2—N1—H1	115.1 (12)	C23—C22—C21	118.32 (13)
C2—N2—C21	126.59 (11)	C23—C22—H22	120.8
C2—N2—H2	115.4 (13)	C21—C22—H22	120.8
C21—N2—H2	117.6 (13)	O2—C23—C24	115.28 (13)
O1—C1—N1	123.98 (12)	O2—C23—C22	123.66 (14)
O1—C1—C11	121.95 (11)	C24—C23—C22	121.05 (13)
N1—C1—C11	114.07 (11)	C25—C24—C23	119.40 (13)
N2—C2—N1	116.58 (11)	C25—C24—H24	120.3
N2—C2—S1	126.15 (10)	C23—C24—H24	120.3
N1—C2—S1	117.27 (9)	C24—C25—C26	120.92 (13)
C12—C11—C16	117.55 (12)	C24—C25—H25	119.5
C12—C11—C1	121.67 (12)	C26—C25—H25	119.5
C16—C11—C1	120.70 (12)	C21—C26—C25	118.84 (12)
C13—C12—C11	122.22 (15)	C21—C26—H26	120.6
C13—C12—Cl1	118.95 (13)	C25—C26—H26	120.6
C11—C12—Cl1	118.82 (10)	O2—C27—H27A	109.5
C14—C13—C12	118.84 (16)	O2—C27—H27B	109.5
C14—C13—H13	120.6	H27A—C27—H27B	109.5
C12—C13—H13	120.6	O2—C27—H27C	109.5
C15—C14—C13	120.57 (14)	H27A—C27—H27C	109.5
C15—C14—H14	119.7	H27B—C27—H27C	109.5
C13—C14—H14	119.7	O2—C27'—H27D	109.5
C14—C15—C16	119.94 (16)	O2—C27'—H27E	109.5
C14—C15—H15	120.0	H27D—C27'—H27E	109.5
C16—C15—H15	120.0	O2—C27'—H27F	109.5
C15—C16—C11	120.87 (15)	H27D—C27'—H27F	109.5
C15—C16—Cl2	119.39 (12)	H27E—C27'—H27F	109.5
			
C2—N1—C1—O1	0.2 (2)	C12—C11—C16—C15	−0.6 (2)
C2—N1—C1—C11	179.99 (13)	C1—C11—C16—C15	−177.50 (13)
C21—N2—C2—N1	−176.37 (12)	C12—C11—C16—Cl2	−179.99 (10)
C21—N2—C2—S1	2.5 (2)	C1—C11—C16—Cl2	3.08 (18)
C1—N1—C2—N2	2.1 (2)	C2—N2—C21—C26	135.10 (15)
C1—N1—C2—S1	−176.87 (12)	C2—N2—C21—C22	−46.8 (2)
O1—C1—C11—C12	−99.98 (17)	C26—C21—C22—C23	−2.4 (2)
N1—C1—C11—C12	80.23 (16)	N2—C21—C22—C23	179.66 (14)
O1—C1—C11—C16	76.83 (18)	C27'—O2—C23—C24	−156.8 (14)
N1—C1—C11—C16	−102.97 (15)	C27—O2—C23—C24	172.8 (8)
C16—C11—C12—C13	0.1 (2)	C27'—O2—C23—C22	22.3 (14)
C1—C11—C12—C13	176.99 (13)	C27—O2—C23—C22	−8.1 (8)
C16—C11—C12—Cl1	−178.93 (10)	C21—C22—C23—O2	−177.57 (17)
C1—C11—C12—Cl1	−2.03 (17)	C21—C22—C23—C24	1.4 (2)
C11—C12—C13—C14	0.4 (2)	O2—C23—C24—C25	179.39 (17)
Cl1—C12—C13—C14	179.37 (13)	C22—C23—C24—C25	0.3 (3)
C12—C13—C14—C15	−0.3 (3)	C23—C24—C25—C26	−1.2 (3)
C13—C14—C15—C16	−0.1 (3)	C22—C21—C26—C25	1.5 (2)
C14—C15—C16—C11	0.6 (2)	N2—C21—C26—C25	179.58 (13)
C14—C15—C16—Cl2	−179.99 (14)	C24—C25—C26—C21	0.3 (2)

### Hydrogen-bond geometry (Å, °)

**Table:**

*D*—H···*A*	*D*—H	H···*A*	*D*···*A*	*D*—H···*A*
N2—H2···O1	0.89 (2)	1.97 (2)	2.7007 (15)	137.9 (18)
N2—H2···O1^i^	0.89 (2)	2.38 (2)	3.1015 (16)	137.8 (18)
N1—H1···S1^ii^	0.852 (18)	2.479 (19)	3.3141 (12)	166.7 (16)

Symmetry codes: (i) −*x*+1, −*y*+1, −*z*+1; (ii) −*x*, −*y*+1, −*z*+1.
